# A Review on Development of Bio-Inspired Implants Using 3D Printing

**DOI:** 10.3390/biomimetics6040065

**Published:** 2021-11-19

**Authors:** Ansheed A. Raheem, Pearlin Hameed, Ruban Whenish, Renold S. Elsen, Aswin G, Amit Kumar Jaiswal, Konda Gokuldoss Prashanth, Geetha Manivasagam

**Affiliations:** 1 Centre for Biomaterials, Cellular and Molecular Theranostics, Vellore Institute of Technology, Vellore 632014, India; ansheed.ar@vit.ac.in (A.A.R.); pearlin.hameed@vit.ac.in (P.H.); wruban1990@gmail.com (R.W.); amitj@vit.ac.in (A.K.J.); geethamanivasagam@vit.ac.in (G.M.); 2 School of Mechanical Engineering, Vellore Institute of Technology, Vellore 632014, India; renoldelsen.s@vit.ac.in; 3 School of Advanced Sciences, Vellore Institute of Technology, Vellore 632014, India; ashwinmallesh281@gmail.com; 4 Department of Mechanical and Industrial Engineering, Tallinn University of Technology, Ehitajate tee 5, 19086 Tallinn, Estonia; 5 Erich Schmid Institute of Materials Science, Austrian Academy of Science, Jahnstrasse 12, 8700 Leoben, Austria

**Keywords:** biomimetic, additive manufacturing, 3D printing, ceramics, polymer, metals

## Abstract

Biomimetics is an emerging field of science that adapts the working principles from nature to fine-tune the engineering design aspects to mimic biological structure and functions. The application mainly focuses on the development of medical implants for hard and soft tissue replacements. Additive manufacturing or 3D printing is an established processing norm with a superior resolution and control over process parameters than conventional methods and has allowed the incessant amalgamation of biomimetics into material manufacturing, thereby improving the adaptation of biomaterials and implants into the human body. The conventional manufacturing practices had design restrictions that prevented mimicking the natural architecture of human tissues into material manufacturing. However, with additive manufacturing, the material construction happens layer-by-layer over multiple axes simultaneously, thus enabling finer control over material placement, thereby overcoming the design challenge that prevented developing complex human architectures. This review substantiates the dexterity of additive manufacturing in utilizing biomimetics to 3D print ceramic, polymer, and metal implants with excellent resemblance to natural tissue. It also cites some clinical references of experimental and commercial approaches employing biomimetic 3D printing of implants.

## 1. Introduction

**Figure 1:**
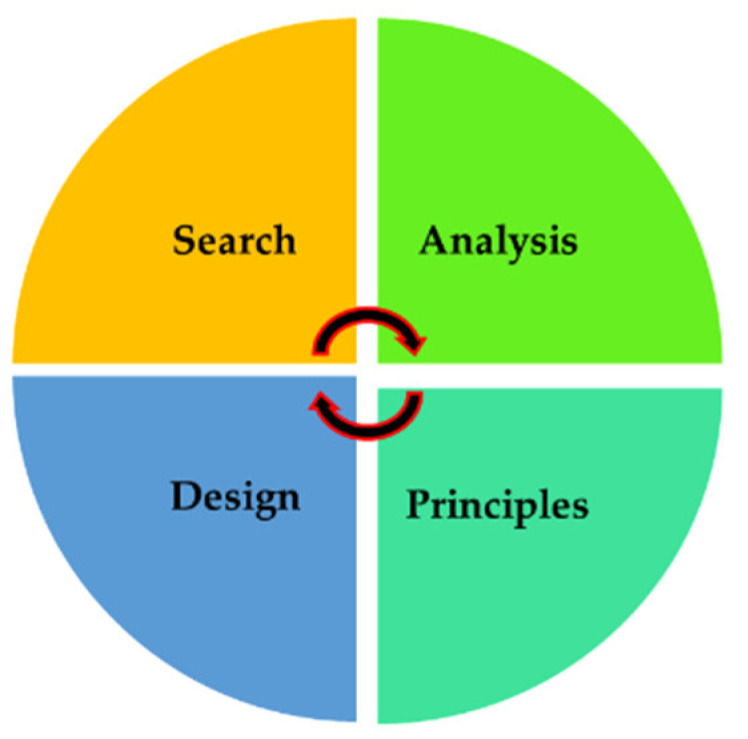
Modus operandi of bio-inspired innovations.

Nature keeps immeasurable innovation and inspirations, which are substantially categorized as visual (shapes and structures) and functional (functionality or multi-functionality) inspirations. Bionic, bio-inspired, bio-mimicry—multiple terms are exclusively owned with regard to nature by borrowing its design or functionality [1]. Derived from a Greek term, the word ‘Biomimetics’ is the imitation of elements, models, strategies, and systems from nature by solving simple to complex human problems. Nature has optimized and enabled organic structure–function relationships with distinguished physicochemical properties. Embracing sustainable mirroring from nature, biomimetics made an interdisciplinary cooperation and technology transfer to other fields that are seeking problem-solving solutions [2]. Many biomimetic principles, ideas, and concepts are applied to real-life applications due to their tenability, efficiency, and elegant design [3]. Bio-inspired tree branch joints (from pine trees) were adopted in aircraft engineering to improve damage tolerance of aircraft bodies using carbon/epoxy composites. Bio-inspired multi-corner tubes copied from bamboo and honeycomb structure are widely applied bio-inspired principles in many engineering problems to enhance the strength of the material without adding any weight [4]. The researchers belonging to a biomimetic group from the University of Toronto expressed four basic research methods for bio-inspired innovations that can be applied to fabricate a product and is depicted in Figure 1 [5]. The ever-growing interest in biomimetics was standardized by ISO 18458:2015. A biomimetic design offers great potential for functional integration, weight-saving and simplifies the design complexities [6].

Additive manufacturing (AM), otherwise popularly known as 3D printing or prototyping techniques, has the ability to transform a three-dimensional digital model into functional components irrespective of geometries [7,8,9]. In the digital manufacturing era, it is possible to achieve ultra-complex structures with enhanced resolution through AM techniques that are extremely difficult to fabricate with conventional machining and molding processes [10,11,12,13]. Tool-less manufacturing, supply chain fractionation, design freedom, and ease of automation are some of the key benefits of AM techniques that revolutionize the manufacturing field and pave the foundation for the rapid growth of this sector. Materials, methods, and energy consumption are efficient in AM, creating an inclination towards sustainability in manufacturing [14,15,16]. AM techniques were introduced to make prototypes, which gradually attained a huge transformation as a reliable direct production technique. The tremendous growth of AM is exhibited in functional component manufacturing for critical applications, such as space exploration, medical device manufacturing, implantology, aerospace development, marine exploration, and defense innovations [17,18,19]. Light-weight structures with improved mechanical strength and other characteristics processed by AM techniques for aerospace and medical applications are crucial [20,21,22]. Dynamic development of AM techniques in the market has led to the development of new AM approaches for customizing the respective applications. Additively manufactured parts offer economic manufacturing with superior design performance, which is appraised as an intelligent part design [23,24]. Various materials, such as metals, polymers, ceramics, and composites in the form of powder, filament, resins, and liquids can be reliably processed by AM techniques [25,26,27]. The fiscal turnover of AM production is obtained through the processing of metals, polymers, ceramics, and others for various applications; among these, polymers are contributing around 80%, and the share of metal printing is also burgeoning due to the biomedical and automotive industry [28,29,30,31,32].

The exponential development of AM and biomimetics can be understood by checking the bibliometrics of published research and review articles in the last ten years. According to the ScienceDirect directory, around 4000 research works concerning ‘additive manufacturing’ and around 3500 research works related to ‘Biomimetics’ were published in 2018. In the last five years, 150 published papers on biomimetics-influenced AM techniques depict that this field is one of the fastest-growing research fields. The mutual integration of biomimetic approaches with AM technologies can exist as solution-driven (biology-to-design) or problem-driven (design-to-biology) [1,33]. In addition to that, freeform design (customized design), simulation-driven design, and lattice design approaches are practiced for AM fabricated components with varying levels of biological input or bio-inspiration made possible. Biological material has great potential and influences AM with its nature-imposed design features, such as fibrous, helical, gradient, layered, tubular, cellular, suture, and overlapping structures. The unique combination, design phenomena, and superior properties of biological components are adopted and employed in various engineering systems on a design or functional basis. AM looks into the design philosophy of green materials, which are considered to be ‘eco-designs’ because they enable the minimum material usage giving more room for sustainable engineering. The distinctive nature of biological materials, such as hierarchy, multi-functionality phenomena, self-healing, self-assembly, and synthesis, render these materials intrinsically different from synthetic materials [34].

Taking the inspiration from biological materials and their structures, the complex geometries and mechanisms, such as gradient geometries, multi-material, and composite structures, can now be reproduced using AM with advanced chemical and synthesis methods [35]. A few interesting approaches include ‘Voronoi diagram’ or ‘Tessellations,’ a generative design approach used for cranial prosthesis designing. Interestingly, a customized and patient-specific cranial implant was designed by mimicking bone trabeculae to reconstruct cranial defects using this approach [36]. Moreover, bio-inspired lattice structures (cellular materials) were employed for an artificial orthopedic hip implant made of Inconel 718 superalloy with a functionally gradient approach [37]. It was observed through finite element analysis that functionally graded lattice structures for the hip implant are appropriate, capable of resisting up to two times the in vivo loads, and are potent to be used as solid implants [38]. When it comes to polymer 3D printing, the thermoset polymer resin is processed by Digital Light Processing for cortical bone reconstruction. Initially, in silico biomimetic models were generated through parametric modeling. From the microstructures, it was understood that the intricate hierarchical structure has acceptable porosity levels [39]. Direct Ink Writing (DIW), an AM approach used to construct viscoelastic ink layer by layer, can be made of hydroxyapatite for engineered bone applications [40].

The central theme of additively-manufactured biomedical constructs is to develop strategies for designing and constructing complex biomedical structures. By embracing a biomimetic approach, AM-based biomedical constructs serve in tissue engineering applications for two broad reasons:

- To replace or repair undermined organs with suitable implanted biomedical constructs.
- To build tissue/organ models for biological analysis and assessment, such as drug screening, toxicity analysis, cell-material interaction.

This review intends to elaborate on additive manufacturing of biomedical implants and their functionalities using polymeric, ceramic, and metallic biomaterials incorporated with biomimetic principles. Clinical trials of biomedical implants illustrated the impact of the biomimetic approach through various case studies and are concluded with future directions.

## 2. Ceramic Based AM with Added Functionalities and Application

Ceramics are considered to be the first explored material for various applications, as their usage date back to 22,000 BC [41]. The multiple classes include oxide and non-oxide ceramics, ceramics composites, glasses, and carbon-based ceramic materials. Alumina, Zirconia, and Silicate are common ceramics that are mostly used in applications requiring high wear resistance, high corrosion resistance, good electrical resistance, and superior thermal stability. Carbon-based ceramic, such as Graphite, Diamond, Graphene, Fullerene, and CNT, are known for their contrast characteristics and diverse applications. Ceramics composites, such as Mullite, Zirconia, toughened Alumina, Carbide Ceramics, and Nitride Ceramics, are also used in manufacturing, biomedical, automobile, and aerospace applications. A separate ceramics band termed bio-ceramics, namely Alumina, Zirconia, Hydroxyapatite (HA), Tricalcium phosphate (TCP), Bio-glass, and Calcium sulfate, are well known for their biomedical applications [42]. The processing of all the above mentioned ceramic material offers great challenges due to its brittleness, least fracture toughness, and high melting temperature. Due to the brittleness and low fracture toughness, the machining or forming of ceramics is not possible under normal conditions. The high melting temperature of ceramics demands the need for a high-temperature facility for obtaining finished products. Higher shrinking also leads to difficulties in achieving the final near net shape products. Nevertheless, with all the demerits, they still outperform other materials, which makes them a more interesting material to work with.

With the advent of additive manufacturing (AM), researchers started developing products made of polymers, metals, and alloys [43,44,45,46]. In the early days, ceramics-based 3D printing was difficult due to the extreme process parameter optimization required to develop the products and their fragile properties [47,48,49]. AM, on the other hand, offers a very high-resolution bottom-up construction of the material, which helps in the extreme control over the process parameters to fine-tune the build. Recently many AM techniques, such as Fused Filament Fabrication (FFF), Digital Light Processing (DLP), Stereolithographic, Inkjet printing, Selective Laser Sintering (SLS), and Selective Laser Melting (SLM), have been adapted for printing ceramics. In addition, a few AM techniques, such as Robocasting [50], Binder Jetting [51], Freeze-form Extrusion Fabrication [52], and Lithography-based Ceramics Manufacturing (LCM) technology [53], are exclusively developed for ceramic printing.

The additive manufacturing of ceramics gained more interest as it has the potential to be developed into biomedical products, mainly implants and scaffolds, which can be made similar to or mimic the actually replaced body tissue. This is achieved by selecting material with identical chemical and physical properties to induce osteoconduction and osseointegration, designing a structure for the scaffold that mimics bone to induce osseointegration, processing, and providing suitable modification in the structure for faster healing. Further, the bio-scaffold should promote vascularization by oxygen transport to cells and the removal of metabolic waste from cells. Thus biomimetic devices fabricated and implanted reduce inflammation and rejection due to immunological responses when implanted in the host. Apart from AM, various conventional manufacturing methods, such as solvent casting/particulate leaching, phase inversion/particulate leaching, ice-templating/freeze-casting, gas foaming, and electrospinning, are also utilized to prepare biomimetic scaffolds. These methods provide biomimetic scaffolds with internal structure, including a pore size between 100 and 1000 microns and porosity up to 90%, resulting in a randomly arranged structure and limited permeability [54,55,56,57].

### 2.1. Ceramic AM Using Biomimetic Designs

The design of implants that can lead to the development of implants or scaffolds with properties matching the tissue is of prime importance for the success of the implants and scaffolds. Each organ or tissue in the human body has a unique architecture, and it is mandatory for the human-made materials to mimic this architecture to perform the function of the replaced organ or tissue and provide an apt environment for the surrounding cells to survive. Biomimetic macro/micro-porous alumina scaffolds were successfully printed, and they exhibited a compressive strength of 29 MPa, equivalent to the natural bone when measured along the direction of aligned micropores. The inference from biocompatibility studies with the MC3T3 cells suggests that alumina scaffolds offer better cell adherence, cell spreading, good cell viability, and less cytotoxicity [58]. Modulus mismatch between the implant and the surrounding tissues is one of the main reasons for stress shielding in implantology. The Young’s modulus human bone ranges from 11 to 20 GPa, whereas the E of implants ranges from 55 to 220 MPA and this understating serves as the basis for implant design using additive manufacturing. Efforts have been made to print triply periodic minimal surface (TPMS)-based scaffold using LCM with alumina. It was also proposed that the open-cell structure, as well as the architecture, offer a conducive cell-microenvironment [59]. Zirconia-toughened alumina 3D scaffolds were printed using the Robocasting technique, and then after sintering, the porous structures were found to have improved flexibility. The human primary osteoblast was cultured on scaffolds and was found to be consistently dispersed over the whole scaffold thickness [60]. Using FDM fabricated mold microstructure, the alumina and β-tricalcium phosphate ceramic scaffolds with controlled microstructures of 300 and 480 microns were developed. This being an indirect approach, the compressive strength of the alumina and β-tricalcium phosphate were reported to be 130 and 1.4 MPA, respectively, for 305 microporous sizes [61]. In the biocompatibility studies, the cells had a good affinity for TCP than alumina structures [61]. From this study, it was well established that mimicking the structure alone will not be sufficient to improve biocompatibility. HA and type-I collagen and HA composite scaffolds have successfully induced osteoblastogenesis and improved mineralization. Moreover, chondrogenesis is found at the center of channeled collagen scaffolds than its outer surface [62]. Apart from mechanical properties mismatch, the surface of current implant materials supports bacterial attachment leading to infection. To combat bacterial infection, a novel electrochemical additive manufacturing method claiming to be cost-effective and time-saving is used to prepare cicada-inspired nanostructured surfaces that are highly antibacterial. The nanopillars structures developed using this technique were found to be closely packed with a diameter of ~65–95 nm and a height of ~380–510 nm, which promote the antibacterial activity of the surface [63]. It is well-established that the nanopillars with 80 nm diameters are lethal to gram-negative and positive bacteria, provided the nanopillar density is significant enough to disrupt the bacterial membrane [64].

### 2.2. Recent Approaches Using Ceramic Biomimetic AM

Hydroxyapatite (HA) is well known and used extensively for medical applications as the chemical constituents resemble the bone composition. Compared to micron-sized HA, the nano HA exhibits superior biocompatibility as it resembles the structure of HA crystals in bone [65]. HA nanorods were synthesized using a hydrothermal technique in a type I collagen matrix to mimic bone composition using a 3D bioprinter. However, the strength of the nanorods was reported to be below average [66]. Yongxiang Luo et al. printed Ca7Si2P2O16 using coaxial 3D printing strategy, where hollow-strut structures of bio-ceramic scaffolds with macropores and multi-oriented hollow channels were designed. The scaffold exhibited high porosity and surface area with remarkable strength. It also had improved cell attachment to the scaffold and better cell proliferation and in the center of the scaffolds, further promoting the formation of new bone [67].

HA scaffolds with defined macroporosity were developed employing a powder-based 3D-printing process and post-treated with polymeric infiltration to mimic natural bone with its elastic collagen structure. This scaffold impregnated with biodegradable polymers, such as gelatin, PVA, and PCL, exhibited superior mechanical properties, good bioactivity, and osteoconductivity. The compressive strength of the HA-gelatin composite was observed to be 12.6 MPa [68]. In a different approach, a microscale mask image projection stereolithography technique was used to develop a HA/TCP scaffold of complex geometry with biomimetic features and hierarchical porosity. The scaffold with 30 wt% HA/TCP with biomimetic hierarchical structure exhibited superior mechanical properties of 4.32 MPa when sintered at 1250 °C with a little over 15% porosity. The live and dead staining studies carried out at 25, and 96 h revealed that this scaffold promotes cell viability as the dead cells were found to be 10% of the total cell population. The scaffold had sufficient strength for replacing the function of critical load-bearing bone. The in vivo study was performed in a nude mouse with a long bone defect carrying cranial neural crest cells and bone marrow mesenchymal stem cells [69].

In another study by Ewhierarchical, a porous HA biomimetic ceramic 3D-printed scaffold incorporated with covalent, modular, controlled-release system (3DPs) housing BMP2 protein to promote osteogenesis was developed successfully 70.The compressive strength was found to be 9.34 MPa. The micro-CT results revealed improved osteointegration and tissue ingrowth. The authors proposed that the scaffold has a therapeutic functionality because of which it can ease graft demand [70]. In another study, a laser-aided gelling (LAG) processing of SiO2-sol mixed with CaCO3 powder was used to develop scaffolds with inter-pore structures. The highest compressive strength of 47 MPa was reported for 5% wt. CaCO3 to SiO2 slurry. It also exhibited bone cell attachment and growth with nil cytotoxicity. It was reported that it exhibited strength–porosity that mimics human bones [71].

### 2.3. Ceramic Processing for Biomimetic AM

The biomimetic hydroxyapatite/gelatin scaffolds are prepared using robocasting of reactive slurries in a bioinspired low-temperature self-setting alpha-tricalcium phosphate/gelatin ink. The composition, crystallinity, and microstructure are closer to the mineral phase of bone, leading to higher reactivity and resorbability than high-temperature sintered hydroxyapatite. The structure has a controlled and fully-connected pore network of 300 µm. A higher specific surface area of 22.88 m^2^/g (for gelatin crosslinked) and 24.85 m^2^/g (non-crosslinked) was observed due to micro and nano-porous calcium deficient hydroxyapatite crystals matrix of needle-shape. The compressive strength was 16.6 MPa for the crosslinked scaffolds, 76% more than the non-crosslinked. However, no significance is found in the stiffness of the scaffolds with 240 MPa for crosslinked and 227 MPa for non-crosslinked. The rMSCs were used, and the cell adhesion and proliferation were found to increase due to the presence of gelatin [72].

Mg-HA composite scaffolds of cylindrical shape were printed using 3D printing. The Mg-substituted HA nanoparticles were synthesized from a bi-template induced biomimetic approach by combining type-I collagen and citric acid. Electron microscopic images revealed that the Mg-HA particles had a plate-like morphology with the dimension of 30–50 nm, similar to human bone. The scaffolds were found to have an inter-connected macro-porous structure with a pore size and strut diameter of 400 μm. The cell adhesion, proliferation, and differentiation studies were performed using MC3T3-E1 cells, which also exhibited promising results. The organic and mineral percent possessed by the human bone was 50–40% and 50–60%, respectively, which is similar to human bone [73]. Three-dimensional-printed HA samples were immersed in 1 M disodium hydrogen phosphate solution for one day at 80 °C, and the influence on biomimetics was cross-checked by assessing the co-deposition by changing soaking time and soaking temperatures, and BSA concentrations. At a 50 °C soaking temperature, the weight reduces after 16 h due to partial dissolution of HA with increased temperature. However, at 23 °C and 37 °C soaking temperatures, the maximum soaking time reached 24 and 8 h, respectively. It was also observed that there was an inverse relationship between the BSA concentration and the density of the scaffold [74]. In a biomimetic deposition technique, HA samples were 3D printed and then immersed in accelerated calcium phosphate solution (ACS). From the micrographs, it was observed that sharp and interconnected plate-like crystals were grown vertically on the surface of needle-shaped hydroxyapatite crystals. It was reported that the biomimetic approach depends on the crystallization in supersaturation conditions, so competition between crystal nucleation and crystal growth is happening that eventually determines the ultimate crystal structure [75].

### 2.4. Ceramic Modifiers for Biomimetic AM

HA is found to have excellent biocompatibility with host cells and does not provoke an immune response. In vitro and in vivo studies have already proven the extent of biocompatibility this compound has with osteoblasts [76] and mesenchymal stem cells [77]. Moreover, implants coated with HA promote early osteointegration and do not create any inflammatory reactions, according to studies [77]. HA nanoparticles combined with PLLA were printed by the FFF method. The HA nanoparticles were modified with dopamine and hexamethylenediamine, and the PLLA chains were grafted on HA nanoparticles by aminolysis reaction. SEM analysis revealed that the rod and hole widths were 300 and 800 μm, respectively. The PLLA/HA scaffolds displayed a rougher surface due to extrusion swelling. The printed structure had high compressive strength (4 MPa), which supports the proliferation and osteogenic differentiation of osteoblasts [78]. In another approach, the β-TCP bone tissue scaffolds are designed with pores as a reservoir of Alendronate (osteoporosis treatment drug) are 3D printed using the binder jetting method, and then coated in PCL for a proper release of the drug. The TCP + Alendronate + PCL performed better than the TCP + Alendronate as there was a significant increase in bone formation [79].

Calcium phosphate is an important bone mineral. The bone is the major elemental mineral reservoir that stores 99% and 80% of the body’s total calcium and phosphate content, respectively, thus making it a suitable compound for developing bio-resorbable products. A 2007 study by Lickorish et al. prepared a third-generation biomaterial exploiting this basic understating, and a polymeric-CaP composite biomaterial was prepared with microporous interconnectivity.

Beta-TCP is reported as the most suitable bio-resorbable material compared to HA and α-TCP as it takes just over a month to degrade [80]. The binder jetting method was employed to process Beta-TCP, which exhibits superior osteoconductive abilities with enhanced osseointegration of complex three-dimensional structures. The osteoinduction property of Beta-TCP is reported to be less, and this is enhanced by doping silica and zinc oxide. The dopants modulate collagen-I and osteocalcin production, which boosted the de novo bone formation and neovascularization by three times [81]. The addition of magnesium and silicon to direct 3D printed scaffolds of TCP promotes in vivo osteogenesis and angiogenesis. The printed structure had a total open porosity of 50.21% after sintering with a pore size of 394 microns, and it exhibited 6.79 MPa compressive strength when sintered at 125 °C for 2h. A significantly higher bone and blood vessel formation are observed for the TCP scaffolds with magnesium and silicon from histomorphology, and histomorphometric analysis than plain TCP scaffolds [82]. By combining inkjet printing and freeze-drying methods, porous silk fibroin and bioactive glass composite were fabricated with interconnected structure and controlled architecture. The two levels of pores in the order of 500–600 µm and 10–50 µm were found in the scaffold. The SF-BG scaffolds prepared with microparticles demonstrated a compressive strength of 1.2 MPa, slightly better than scaffolds prepared with nanoparticles. However, the scaffold with nanoparticles presented a 50% hike in the attachment of human bone marrow stem cells compared to microparticles incorporated scaffold [83].

Thus, the 3D printing of ceramic scaffolds is revolutionizing biomaterial manufacturing; however, the design of the biomimetic scaffold depends on factors, such as gender, age, bone defect (size and kind), structural behavior, biochemical stimuli, vascularization, inflammatory and immunological processes, expected during bone regeneration. It should be manufactured based on the type of cells and tissue morphologies of the bone tissue to be restored, thus gaining natural functionalities and displaying ideal biochemical and topographical traits to permit the infiltration of MSCs and other osteoprogenitors cells [84,85].

## 3. Polymer-Based AM with Added Functionalities and Application

Polymers are processed by various AM techniques with added biomimetics approach substantially benefitted for various biomedical applications. Various polymer materials, such as single entity, multi-materials, polymeric composites (with metals, ceramics, and polymer-polymer blends), fillers, coating materials, and functionally graded polymers, are predominantly used for orthopedic bone and dental applications. Apart from these, polymeric nanostructured materials are applied in drug delivery, gene carrying, bioimaging, tissue engineering, and regenerative medicine applications [86]. By promoting biodegradable and recyclable polymers, environmental sustainability could be maintained. Specifically, PLA (Poly Lactic Acid), a popular polymer material that is made from the polymerization of sugars and starches, and polyhydroxyalkanoates (PHA), from sugars with biosynthesis used in Fused Deposition Modelling (FDM), can keep the sustainability criteria. These bio-polymers produce fewer fumes and smells than synthetic petroleum-based polymers. In addition to that, adding bio-based fillers can improve sustainability without harming nature. Filler can be fibers or particles, such as sawdust, thermoplastic starches, cellulose fibers, or other natural fibers: bamboo, birch, cedar, cherry, coconut, cork, ebony, olive, pine, or willow reinforced with synthetic polymers to form biocomposites [87]. For instance, lignin, a biomass material from industrial feedstock waste, was added with synthetic polymers acrylonitrile-butadiene-styrene (ABS) and nylon 40–60 wt.% to promote sustainable development manufacturing. The lignin-based bio-composite exhibits upgraded tensile strength and stiffness at room temperature processed by extrusion-based AM technology called FDM [88]. Sustainable vascularized microtissues were 3D printed for soft tissue repair in vivo as self-assembled building blocks. Aside from tissue repair, such constructs can be used as sustainable in vitro disease models [89].

**Figure 2:**
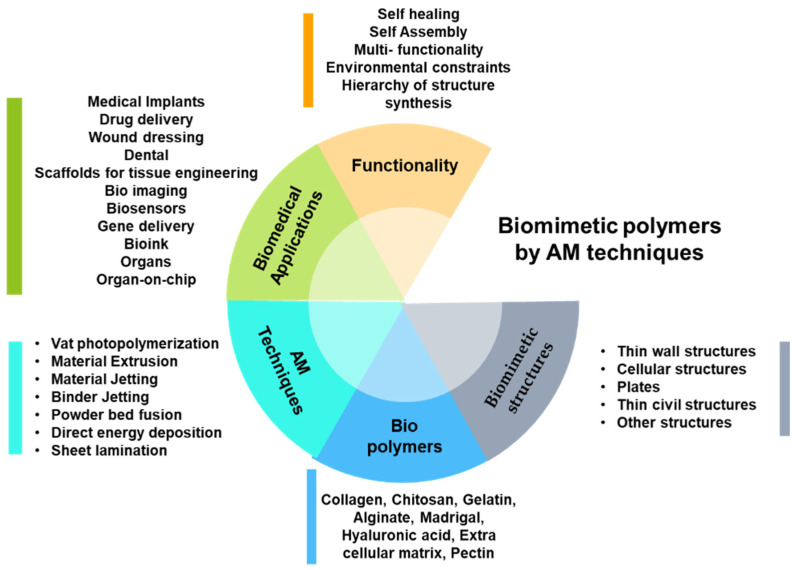
Polymers with biomimetic functionalities processed by AM techniques.

The elastic nature of intervertebral disk (IVD) tissue was mimicked, and the artificial scaffold was fabricated using the extrusion-based AM technique with degradable polyurethane material. The binder jetting AM technique was adopted to produce load-bearing, porous metallic bone scaffolds, which mimic lamellar plywood motifs for tissue engineering applications [90]. A custom-designed degradable polyurethane (PU) construct was made with the biomimetic elastic nature of the native IVD tissues using an extrusion-based bioprinting technique. The constructs exhibit good elastic nature under compression, similar to native IVD tissues. Precise control over cell morphology, an ideal condition for cell attachment and ECM deposition, and the capability to produce large quantities of tissue scaffolds are key advantages of these material–method combinations [91]. Three-dimensional helicoidal structure mimicking the structure found in mantis shrimp was used to develop lab-scale 3D biomimetic tough helicoidal structure using polycaprolactone (PCL) and polyvinylidene fluoride (PVF) fabricated through novel near-field electrospinning technique (NFES). The results showed that bio-inspired helicoidal structures provide a high load-carrying capacity and better crack and delamination resistance [34]. Figure 2 represents the polymeric components used in biomedical applications fabricated by AM techniques with biomimetic functionality.

Alginate is used as a hydrogel for the bio-fabrication process. It has strong mechanical properties for ideal bio-fabrication. In another study, alginate was combined with nano-cellulose and used as a bio-ink [64,65] to assess the biocompatibility with human nasoseptal chondrocytes. An extrusion-based 3D bioprinter printed this combination with optimized process parameters. Alginate–nano-cellulose bio-ink demonstrated post-printing shape fidelity (reversible stress softening behavior), a high degree of shear thinning, and a stable construct volume [66]. Photopolymerizable polymer (composed of urethane acrylate oligomers) was designed with cellular structures with four precisely controlled internal architectures (octahedral, cubic octahedral, pillar octahedral, and truncated octahedral) for biomimetic bone implants. The implants were fabricated by 3D printing technologies and evaluated for their biological and mechanical behavior. Among the four internal architectures, it was found that the pillar octahedral had balanced mechanical and biological properties [92]. The evolution of nature-derived polymer-based new products with a biomimetic approach yields sustainable development in additive manufacturing. Table 1 represents the biomimetic polymers used in functional biomedical applications fabricated through AM techniques.

Hybrid AM processing was amended for dental applications using metal and polymer materials. The SLM process was used to produce a metal mesh of dental implant, and it was placed in the SLA platform. The photosensitive polymeric resin fills the core and coats around the tooth for the teeth crowns. The metal-polymer gradient multi-material successfully manufactured by SLM/SLA hybrid AM techniques by adopting the biomimetic approach exhibits good biomechanical strength. This approach was espoused to manufacture bio-inspired patient-specific dental implant cores using metal, ceramic, polymer, or composites, which exhibited superior performance. Furthermore, dental crowns are coated with polymeric materials to obtain good physicochemical and biomechanical characteristics [93]. The feasibility of mimicking the natural curvature and dimensions of human cell membrane models, i.e., red blood cells, smooth muscle cells, and columnar epithelial cells with microvilli, has also been assessed. These cells have been chosen with distinctive features, shapes, and sizes and produced through two-photon polymerization additive manufacturing using polymeric cell scaffolds and coated with the cationic polymer PLL (Polylysine). The model can reproduce human cell-like shapes with high fidelity, suitable for incorporating transmembrane proteins and useful tool for drug delivery studies [94].

**Table 1:** Polymer, biomimetic functions, AM techniques, and biomedical applications.

Polymers	Biomimetic Functionality	Biomedical Application	AM Processing Technique	Advantages
**Natural polymers (proteins and polysaccharides)**				
Collagen [95]	Biomimicking native tissue	Skin replacement, hydrogel, bioink	Extrusion, Fusion	Mechanical stiffness, viscosity, biodegradability
Gelatin [96]	Biomimicking native tissue	Bioink, hydrogel	3D Bioprinting, SLA, extrusion, inkjet printing, 4D printing, Laser printing	Biocompatible, biodegradable, flexible
Chitosan-HAp (hydroxyapatite) [97]	Biomimicking native tissue	Hydrogel, scaffolds, bone tissue engineering	3D printing	Biocompatible, cell viability, cell-friendly environment, adequate mechanical properties
Hyaluronic Acid [98]	Biomimicking native tissue	Hydrogel in cartilage regeneration	3D printing	Superabsorbent, cytocompatible
Alginate [99]	Biomimicking native tissue	Bioink, hydrogel	Bioprinting, extrusion printing	Cell-protective effect, cell viability
Silk Fibroin [99]	Biomimicking native tissue	Scaffold, bioink	Bioprinting, extrusion printing	Superior mechanical properties and tunable degradability
Fibrin ink	Biomimicking native tissue	Vascular constructs	Inkjet printing	[100]
**Synthetic polymers**				
Polycaprolactone (PCL) [101]	Biomimicking native tissue, multi-functionality	Tissue engineering wound dressing	FDM	Low melting point, Biocompatible
Polyurethane (PU) [91]	Biomimicking native tissue	Tissue engineering, prosthetic devices	Binder Jetting, FDM	Elasticity
Polyether ether ketone (PEEK) [102]	Biomimicking native tissue	Dental, orthopedic, trauma, and spinal implants	Laser printing, extrusion	Superior mechanical properties, inert, biocompatible
Polyethylene glycol [103]	Biomimicking native tissue	Porous scaffold, Implants, Drug delivery	Extrusion	Biocompatible
Polylactic acid (PLA) [104]	Biomimicking native tissue	Scaffolds, prosthetic devices	Extrusion based bioprinting	Mechanical strength
Acrylonitrile butadiene styrene (ABS) [105,106]	Functional models	Prototypes, cost-effective prosthetic devices	Extrusion	Low cost
**Polymer composites**				
Polyethylene glycol (PEG) derivatives mixed with fibroblasts [107]	Hollow tubular structures	Vascular constructs	Extrusion	Biocompatible
Polyglycolic acid/polylactic acid (PLA/PGA) scaffolds [108]	Native stiff bone-like constructs	Cartilage–Bone	3D Printing	Biocompatible, stiffness
polyethylene glycol (PEG)/β-tricalcium phosphate (β-TCP) scaffold [109]	bio-inspired interface structures	Cartilage–Bone	Stereolithography	Biocompatible
PLA/HA screw-like scaffold [110]	Native bone	Bone	3D Printing	Bio-active, mechanical properties
Chitosan-based polymers (N-succinyl chitosan grafted polyacrylamide [111]	Shape memory function (pH)	Drug delivery; Bone regenerative therapies	4D Printing	Biocompatible, Better controlled release of drugs; Tunable mechanical properties
Gelatin-polycaprolactone (PCL) [112]	tubular structures	Bilayers, Cell-laden bioscaffolds for tissue engineering	4D Printing	Compatible; Biodegradable
Poly-ethylene glycol (PEG) [113]	Shape memory function (Humidity)	Cell-laden bilayers	4D Printing	Biocompatibility

## 4. Metal-Based AM with Added Functionalities and Application

Implantology dates back to the pre-Columbian era, with some of the early mentions around 2500 BC regarding the use of gold ligatures for teeth stabilization [114]. Since then, metal has been the most preferred material for load-bearing implant construction, other than early implant materials, such as ivory, rubber, and even animal bone [115]. Metals are inert, biofriendly, have good strength, higher fracture toughness, wear, and corrosion resistance, decent fatigue-proof hardness, and excellent for all load-bearing applications [116]. Metallic biomaterials were considered a suitable replacement for treating bone defects. The most commonly used metallic biomaterials are constructed using stainless steel (SS), cobalt alloys, magnesium alloys, pure titanium, and its alloys with vanadium, nickel, silver, tantalum, and zirconium [117,118,119,120]. However, all conventional metal implants were poor in mimicking the native properties of human bone, leading to further complications in the long run. One of the major drawbacks is their surplus elastic modulus, which leads to stress shielding, a phenomenon in which implants with high elastic modulus and tensile strength (such as Titanium or SS) create an uneven bodyweight distribution, leading to bone loss or osteopenia over the long run [121]. Resorbable elements, such as Iron or Magnesium, get absorbed into the body, causing premature weakening or loss of implant strength [122]. Alloy implants, such as Ti-15Zr-10Cr or Ti-6Al-4V have been found to release vanadium or chromium ions into human cells, leading to cytotoxicity [123]. Some of these challenges can be addressed if the choice of material and its processing could give a more biomimicking implant that deviates very little from the natural organ that is being substituted.

It has been observed that the success rate of any implantation is highly dependent on biomimetics. Say, for example, the healing of bone through direct or indirect mechanisms is a complex biological process, and a bone implant substituting a defect has to be designed in such a way that it should promote natural bone healing by triggering mesenchymal stem cells recruitment, generation of callus, revascularization, neoangiogenesis, mineralization of new bone, and finally remodeling [124]. Fortunately, the research world is putting efforts into addressing the abovementioned aspects during the manufacturing stage, and additive manufacturing (AM) is a promising approach with very high biomimetic capability. Additive manufacturing (3D printing or rapid prototyping or rapid manufacturing) is a three-decade-old bottom-up fabrication approach that is currently in the vanguard of biomaterial processing. Metal AM is found to be an efficient processing technique to fabricate implants with very high precision and patient specificity. The next section will substantiate the importance of AM in promoting biomimetics in implants.

### 4.1. Importance of AM in Construction of Biomimicking Implants

AM is an emerging material processing technique that can revolutionize implantology, especially for hard implants. Since this is a computerized bottom-up technique, there is fine control over the anatomical features of the lost tissue and can even imitate the micro and nano-level topographical features of the bulk material used for implant processing. Usually, the site of injury is scanned using Computer Tomography (CT) or Magnetic Resonance Imaging (MRI) to obtain Digital Imaging and Communications in Medicine (DICOM) data, and with the help of CAD or modeling software, the replica of the lost tissue is designed (cross-check this). The 3D printer stacks the metal feed in a series of layers based on the software input to create complex and patient-specific structures. This facile processing technology made it much more cost-effective than the conventional approach and made it a burgeoning orthopedic repair mechanism. There are various materials used in implanting, of which metals are best suited for the load-bearing application, thus becoming the gold standard for orthopedic repair and corrections. AM allows the existing metal modeling for biomedical applications to be more bio-friendly and mimicking.

#### 4.1.1. Mimicking Mechanical Properties of Natural Bone

Apart from the technological superiority of the process, AM helps overcome the two important shortcomings with the conventional bio-inert implants, i.e., poor osteointegration and stress shielding. A study by Sajad et al. had clearly demonstrated the importance of implant architecture and dexterity of AM in overcoming this limitation [125]. AM mimics the natural architecture of the human bone, thanks to the computer-assisted layer-by-layer stacking with precise control over the size and porosity of the implant material. This porous nature of the material reduces the elastic modulus of the implant, making it comparable to that of natural bone, thus preventing the stress shielding effect [126]. In a 2015 study by G. Rotta et al., it was demonstrated that SLM-printed porous Ti-6Al-4V had an 85% reduction in elastic modulus. The conventional alloy had a modulus of 110 GPa, while the 3D-printed porous Ti-6Al-4V showed a range of 17–49 GPa, which stands closer to actual human bone, varying from 0.15 to 18.1 GPa [127]. A study by Hasan et al. suggests that shell thickness and porosity parameters for AM is also an important aspect for ensuring adequate mechanical properties [128]. A comparison of the tensile strength and elastic modulus of the natural bone, conventional bone implants, and AM porous implants is given below in Table 2 [96,116,129,130,131,132,133,134]. Three-dimensional printing allows the user to have fine control over mechanical parameters, such as cross-sectional shape and rod diameter, which can influence the porosity, which is an important parameter necessary for biomimicking the actual bone. It ensures the circulation of necessary nutrients, oxygen, and other biological components to initiate bone healing post-implantation. It has been observed that a pore size ranging from 300 to 800 µm is the ideal porosity for mimicking human bone [135]. It has been observed that changing porosity and microstructure can influence the regeneration of bone [136,137,138].

**Figure 3:**
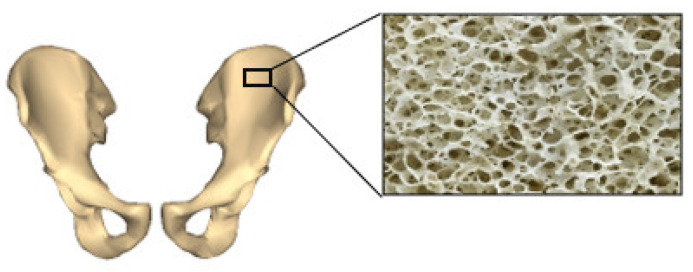
Porosity in natural bone. Anatomography, CC BY-SA 2.1, JP [139] | Patrick CC BY 2.0, USA [140].

**Figure 4:**
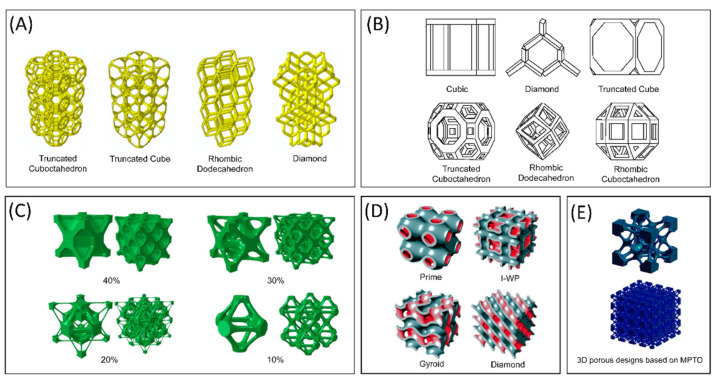
Commonly used unit cell structures for mimicking bone architecture in additive manufacturing. (**A**) Polyhedral circular geometry (**B**) Polyhedral square geometry (**C**) Bidirectional evolutionary structural optimization (BESO) based unit cell geometry (**D**) Triply periodic minimal surfaces (TPMS) based geometry (**E**) Multi-phase topology optimization (MPTO) based three-dimensional porous structures. Adopted, recreated, and reprinted under Creative Common CC BY license from, https://www.mdpi.com/2075-4701/9/9/1004/htm [136].

One of the interesting features of AM is its ability to control and design the lattice structure and gradient structure, which has a critical role in determining the success rate of the implant inside the body. The arrangement of unit cells in a periodic fashion was effective in predicting the performance of the implant and improving the reproducibility and ease of manufacturing. Some of the commonly used lattice structures are the polyhedral models (circular or square), bidirectional evolutionary structural optimization (BESO), multi-phase topology optimization (MPTO), and triply periodic minimal surfaces (TPMS), which stand close to human bone’s lattice arrangement. Natural bone has a varying lattice arrangement throughout its length, as shown in Figure 3. Focusing on lattice parameters alone is not necessary to mimic the actual bone; therefore, in the latest effort, researchers focus on functionally graded materials (FGM), which can accommodate varying lattice arrangements and have adaptive porosity. Figure 4 demonstrates some of the common unit cell structures that can mimic the porosity of natural bone.

#### 4.1.2. Surgical Planning

AM is a simple technique that can create 3D models and guides for surgical planning, which helps doctors make a spot-on analysis pre and post-implantation. Since the design aspect is computer-aided, the surgeon gets a tactile and in-depth understanding of the complex fracture pattern that occurred to the bone. Unlike traditional implantation procedures, the surgeon can have a readymade patient-specific implant model in hand for pre-operative review to plan for intraoperative complications, which is crucial in surgery planning [141,142]. Some of the projected benefits of this approach are improved patient communication, shorter operative time, higher precision in the alignment of components, and a 3D model that acts as a patient-specific reference that improves the safety of the procedure [143]. Three-dimensional models of bone are currently used in the surgical planning of upper and lower limb osteotomies and the correction surgery of diseases, such as spine scoliosis, Blount’s disease, and Perthes disease [144,145].

### 4.2. Conventional Metal Processing and Its Biomimetics

In earlier days, bone defects were repaired using autografting from the cranium, tibia, rib, scapula, sternum, fascia, or ileum were used [126]. In later approaches, allografts (of cadaveric origin) and xenografts from animals were used for cranioplasty [127,128]. However, maintenance, revision surgery, and graft rejection are some of the problems with the above techniques [126]. This led to the use of non-metallic and metallic allografts in rural surgical practice. The bone defect was fixed in the conventional approach using a metal plate or mesh, synthetic bone substitute, or prefabricated bone replacements [129]. Metal allografts made from gold and silver were early contenders, but later, other metals, such as aluminum, tantalum, stainless steel, lead, platinum, vitallium, ticonium, and titanium, were used [126,130,131,132]. However, most of these metals had several mechanical and chemical issues to be used as an implant. For example, pure metals were prone to oxidative or galvanic corrosion, while common metals, such as stainless steel, had high stiffness leading to stress shielding and other inflammatory response due to the conductivity of the oxide layer [133,134]. Due to the excellent biomimetic capability and biocompatibility, titanium alloys have come up as a suitable implant choice [134].

Conventional metal processing techniques include machining forged titanium alloys according to orthopedic design needs. However, this technique results in a lot of material wastage, and the incorporation of complex anatomical design features is not possible with machining [135]. Precision casting is another favorable technique, but the high melting point of titanium (>1700 °C), fluid properties, and faster oxidation make this a challenging processing technique. Some advanced techniques, such as centrifugal precision casting, have been devised but are not an economically viable choice to manufacture porous implants to mimic natural bone architecture [136]. Deep rolling and cold rolling is considered effective alternative against shot-peened titanium; however, rolling techniques are highly limited by their shape choices [137]. Friction stir processing (FSP) is an advanced manufacturing technique in which localized plastic deformation is induced to change the mechanical properties of metal. FSP processed Ti-6Al-4V with surface modifications shows improved cellular adhesion; however, it also has drawbacks similar to conventional techniques [138]. The bulk mechanical property is not changed to match the natural elastic modulus of bone.

All existing implant manufacturing processes can fabricate the bulk material; however, the crux of biomimetics is a combination of biocompatibility, bioactivity, and immune acceptance. The first and foremost requirement for biomimicry is to choose a material accepted by the immune system. Once that is taken care of, the next is to mimic the physical architecture of the tissue (bone) to house the biochemical components. All conventional techniques fail in the second part, where the processing limitations prevent them from copying the actual physical and mechanical properties of natural human bone. This is where additive manufacturing of hard metals gains its superiority. Since the implant is constructed layer-by-layer, there is room for copying the micro or nano-architecture of real bone. The level of detail is currently limited to some of the basic unit cell designs as discussed above but is expected to incorporate the natural design in the near future. Incorporating bioactive elements, such as Zn, Sr, Mg, Nb, during the alloying process is the first step towards making the implant bioactive [139].

### 4.3. Techniques Used for Metal Powder Bed Fusion

Out of the vast plethora of Additively manufacturing (AM) techniques, metallic printing occupies a substantial fraction in the 3D printed market, as reported by a market analysis report 2020 [146]. The automobile, aerospace, healthcare, and consumer products are major sectors exploiting additive manufacturing products to the fullest. The espousal of metal AM technology, mentioned in Table 3, such as Selective Laser Melting (SLM), Electron Beam Melting (EBM), Direct Metal Laser Sintering (DMLS), Laser Engineering Net Shaping (LENS), and Directed Energy Deposition (DED), by manufacturing industries has enabled the rapid fabrication of tailor-made components and products. AM has also reduced the factory to market time. These contemporary techniques give a tough competition to the conventional fabrication techniques, such as casting, forging, and metal injection molding.

**Figure 5:**
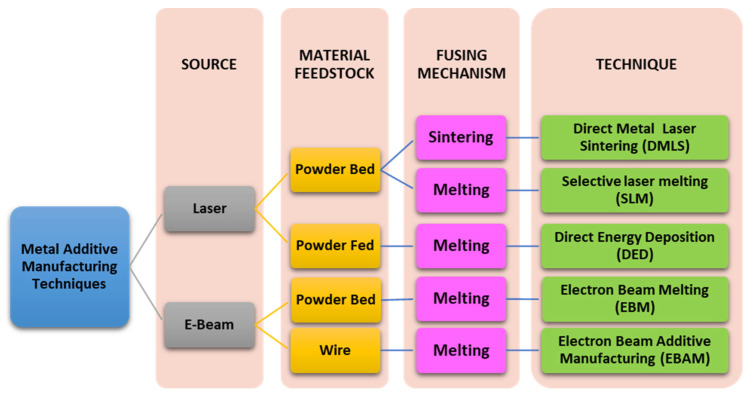
Different metal additive manufacturing techniques.

An overview of the different AM techniques used for metal printing is shown in Figure 5. Each of these additive manufacturing techniques, SLM, EBM, DMLS, and LENS/DED, differ slightly from one another. Still, most of these techniques have a common factor: laser and a similar powder recoating mechanism, except in EBM, which uses electron-beam. Briefly, in all metal additively manufacturing techniques, a laser or an electron beam melts the powder metal particles to fuse the molten metal for a structure layer by layer. The following powder layer spreading mechanism onto the substrate or build plate differs from technique to technique. For example, in SLM, DMLS, and EBM, a re-coater/wiper spreads the powder, while in the case of DED/LENS, the powder is emitted from a nozzle reducing the wastage of powder. The powder is applied and melted, and this process is repeated slice by slice until the part is entirely printed.

**Table 3:** A brief overview of different metallic additive manufacturing techniques.

Technique	Description
EBAM	Among the other metal AM techniques, EBAM can be regarded as a faster and more cost-effective process, mainly due to its wire-feed system. The wire-feed system eliminates the wastage of powder and works faster by eliminating the powder’s recoating time. EBAM with a dual wire-feed nozzle has the added advantage of mixing two different metal alloys during the fabrication of a part, alternating between two different types of metals, and changing their ratio. At the same time, printing can be easily achieved in EBAM. This feature is not achievable in metal powder-bed and powder-fed fusion techniques due to the possibility of contaminating the part with unwanted metal powder.
LENS/DED	LENS systems use directed energy deposition (DED), where high-powered lasers build structures layer by layer directly from powdered metals, alloys, ceramics, or composites to produce fully-dense parts. DED has a coaxial laser and powder emission orifice, which often has inert gas, such as argon or nitrogen blown to sheath the melting region to prevent metal oxidation. This results in a high-speed, high-quality, affordable metal 3D printing process making complex metal parts easier, more precise with excellent mechanical and fatigue properties, and efficient and affordable to produce and repair.
DMLS	The wastage of powder in DMLS is fairly less than methods that involve widespread powder recoating using wipers. A benefit of using DMLS fabricated parts is that objects produced through DMLS do not possess any residual stresses or internal defects. However, the downside of such a high-end technique is its costly production and maintenance cost.
EBM	EBM is very similar to selective laser melting and produces dense and porous parts. The difference between the two techniques is that EBM uses an electron beam rather than a laser to melt the metal powder. Due to the use of an electron beam, the process takes place in a vacuum rather than an inert atmosphere, as in SLM. Another difference between SLM and EBM is that the powder bed in EBM can be pre-heated to up to 700 °C by the defocused electron beam. This plummets the temperature gradient by reducing the rapid heating and cooling.
SLM	SLM involves a high-powered laser that fully melts the metal powder particles and welds them together by melting, giving rise to more robust and denser objects than metal sintering techniques. The powder is heated to a temperature above the metal’s melting point for binding metal particles in a molten state. This rapid heating and cooling process gives rise to a broader temperature gradient, resulting in stress and dislocation in the final product, compromising the product’s mechanical properties. As powder flowability plays a vital role in SLM, only optimized metals are currently being fabricated, such as stainless steel, titanium alloys, chromium cobalt, and aluminum [46,147,148,149].

### 4.4. Post-Processing of AM Products

**Figure 6:**
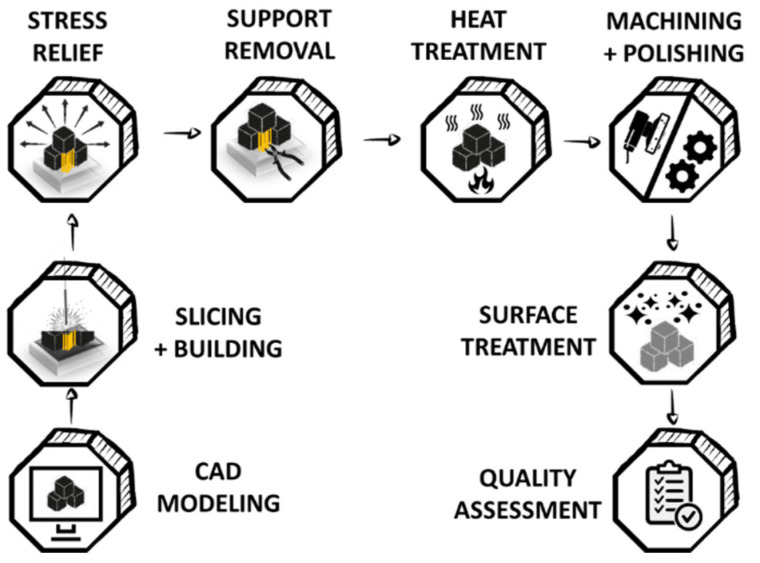
Schematics showing the post-processing workflow.

The melting of metallic powder at high temperatures during different additive manufacturing techniques makes it unfeasible to add bioactive and organic elements during the process. The probability of these elements losing their property while getting melted or welded during the manufacturing process is inevitable. Thus, a 3D printed object has a greater chance of modifying/functionalizing during post-processing than in situ processing. The post-processing workflow is depicted in Figure 6. The first step to post-processing of the manufactured product involves stress relieving. The recurrent cooling and melting of metal powder at a rapid rate cause internal stress build-up within the built product, which must be relieved before the part is separated from the build plate. The presence of residual stress in the final product can lead to distortion and, in extreme cases, might lead to the formation of cracks while intrinsic properties, such as fatigue and tensile strength, are also affected [150,151]. The common method for reducing the residual stress before fabrication involves pre-heating feedstock powder and substrate and changing the scanning strategy [152]. In comparison, the post-built method involves annealing the part in an inert atmosphere. Alternatively, researchers have also developed an in situ method for reducing stress during fabrication by illuminating the building part with homogeneous intensity emitted by laser diodes. This process decreased the large thermal gradient, resulting in a 90% reduction in residual stress [151].

The second step of post-processing involves removing support structures to extricate the parts from the build plate. This can be done manually with the help of pliers or using machines, such as bandsaw and wire EDM. This is followed by heat treatment of the built parts to modify the mechanical properties (yield strength, corrosion resistance, ductility) and rearrangement of microstructure and phase distribution [153,154,155]. ASTM F3301-18a provides a detailed guideline and standard for thermal post-processing methods. This step is followed by machining and polishing, which allows modification of the built product to give a more finished look. It involves removing internal support structure, grinding and smoothing, adding treads, chemical and electrochemical polishing, deburring using centrifugal disc finishing or water jet blasters, abrasive blasting, and shot peening ultrasonic cavitation abrasive finishing, and CNC and vibratory finishing [156,157,158].

### 4.5. Various AM Biomimicking Metal Implants-with Clinical Case Studies

**Figure 7:**
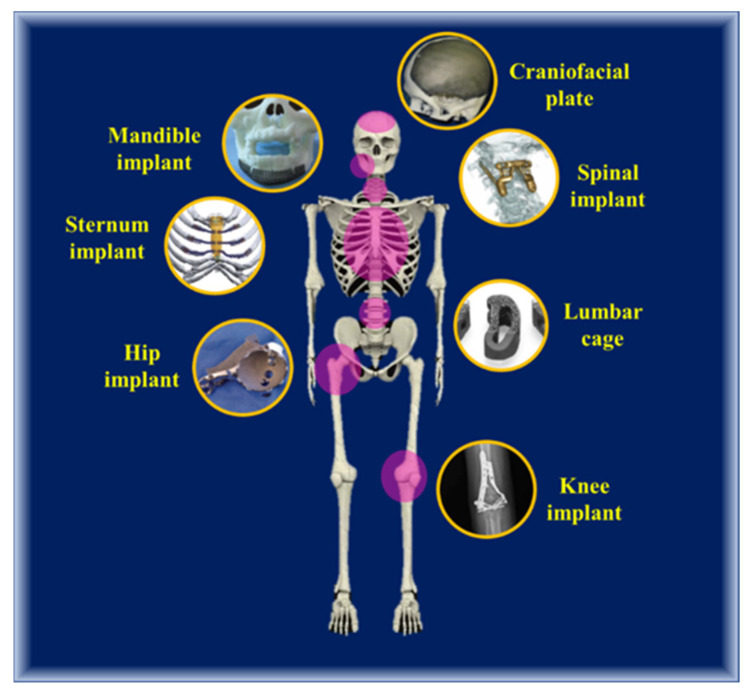
Craniofacial plate implant Ti-6Al-4V DMLS [161], mandibular implants [162], spinal [163] and sternum [164] implants, lumbar cage [165], hip [166], and knee implants [167]. Reprinted under copyright CCBY-NC-ND 4.0 license.

Undoubtedly, metal 3D printing is the best alternative for constructing load-bearing biomimicking implants that can perform on par with natural bone in elastic modulus, compressible and tensile strength, lattice arrangement, and pore size. They are extensively used in the current clinical setup, mostly due to their customizable nature and patient specificity, which, when coupled with the technical superiority of the processing technique, makes it the future of implantology. Metal additive manufacturing of biomimicking implants (as shown in Figure 7) is a decade-old technology, and one of the first reported instances of experimental implantation was performed in 2014 in a 71-year male patient suffering from grade 2 chondrosarcoma. The subject received a custom additively-manufactured titanium heel prosthesis with a smooth surface attaching to the bone tissue, provisions for suturing the implants onto the heel, and a micro-rough surface to promote tissue adhesion [159,160]. The sections below will substantiate the types of biomimicking implants and prostheses and their clinical status, processing technology. Different AM implants and types of processing, the material used are summarized in Table 4.

#### 4.5.1. Cranioplasty Implants

Cranioplasty is the surgical regimen that focuses on repairing skull bone defects that are either caused by accident or previous surgery. Titanium/its alloy as a mesh or dense metal, synthetic bone substitute in liquid form, or solid biomaterial made of metal or polymer is the most widely used clinical materials for the repairs [168]. One of the earliest known rapid prototyping of skull plates was in the early 1990s, where the skull defect was repaired using a stereolithographic printed acrylic model to custom-make a titanium implant to fit the patient’s defect [169]. However, with the advent of metal 3D printing, efforts have been made to print porous skull implants directly using direct metal laser sintering (DMLS) [170]. Another interesting approach was demonstrated by Alida et al. in a 2008 study, where PHANToM 1.5 haptics were incorporated with electron beam melting (EBM) for automated custom printing of skull plates with 50% reduced time consumption [171]. Titanium used to be the favorite metal for prototyping, which Ti-6Al-4V gradually overtook due to its superior mechanical properties. A 2018 study by QichunRan et al. observed that selective laser melting (SLM) printed implants with a pore size of 700 µm exhibited superior bone ingrowth, which points towards the advantage of AM in mimicking natural bone [172]. AM has found applications in designing, simulating, and manufacturing cranial implants. One of the best instances is demonstrated in a 2018 study by Antonio Marzola et al., where a 3D printed model of the defect helped surgeons carefully plan for the surgery to avoid skin flapping complications while an EBM processed Ti-6Al-4V skull implant was used to repair the defect [173].

#### 4.5.2. Vascular Stent

A vascular stent is a mechanical structure secured inside a coronary artery to create a channel for the uninterrupted flow of blood. It is mainly used to treat coronary artery disease or peripheral artery diseases. Percutaneous coronary interventions, balloon angioplasty, and coronary balloon dilation are some clinical practices that make use of a vascular stent [174]. During the early phase of stent implantology, bare-metal stents (BMS), drug-eluting stents (DES), and bioresorbable stents (BRS) are some of the prevalent iterations [175]. Traditional manufacturing practices include photolithography-based etching, micro-EDM, electroforming, die-casting, and laser cutting [175]. Electrospinning has emerged as a novel processing technique for the vascular stent, but with its superior precision for millimeter thick stents, AM is one of the most preferred choices. Stereolithography (SL), selective laser sintering (SLS), and fused deposition modeling (FDM) are the preferred AM methods for stent construction. Demir and Barbara, in a 2017 study, had employed SLM for manufacturing a CoCr stent, which had demonstrated satisfactory mechanical properties and could be a suitable substitute for laser-based micro-cutting [176]. Nitinol is one of the preferred alloys for stent construction due to its superior pseudoelasticity, and it is made by combining varying weight percentages of Titanium and Nickel. A team of scientists from the Commonwealth Scientific and Industrial Research Organization (CSIRO) was the first to 3D print a Nitinol stent using SLM technology [177]. This 3D printed stent undergoes a phase transformation at sub-human body temperature for self-expansion, making it a smart biomaterial unheard of until then. However, 3D metal printing also exhibits certain design constraints, as observed by McGee et al. in a 2021 published study. The SLM-printed titanium stents need to be self-supporting, which creates a design constriction. It was eliminated using a hybrid approach of combining SLM with etching to create open cell designs [178].

#### 4.5.3. Bone Fixtures

AM is widely used to construct bone implants, such as the knee, hip, clavicle, and cranium; however, their potential in custom printing complex fixtures is underexplored [179]. Three-dimensional printed guides for superior contouring were the initial products manufactured using AM, but recent advances in rapid prototyping enable 3D printing of the fixtures. The most commonly used fixtures are bone plates, screws, nails or rods, and wires to immobilize the bone segment that underwent fracture [180]. A 2016 study by Smith et al. describes the clinical use of SLM-printed Ti-6Al-4V extra low interstitial (ELI) bone plate to fix hallux valgus (bunion) deformities, which otherwise would depend on surgical interventions, such as osteotomy [181]. Bone screws are one of the load-bearing fixtures, and bioabsorbable screws are prone to breakage, as reported in several studies, thus demanding the need for metal screws [182,183] In a recent study by Yu-Min et al., an SLM-printed bone screw using Ti-6Al-4V was implanted in a rabbit model to assess the feasibility of the interference screw fixation method. The screws were surface modified chemically using calcium phosphate to improve the bioactivity of the screw-bone interface [184]. Another customized intervention was reported by Ying Zhang et al., where a Ti-6Al-4V trabecular bone and connecting rods were printed using electron beam manufacturing technology for treating early-stage osteonecrosis in the femoral head [185]. A customized clinical intervention is very uncommon in bone pathophysiology and made possible only because of AM.

#### 4.5.4. Hip and Knee Implant

The hip, knee, and ankles joints are the most load-bearing intersections in the human body. One of the major hurdles with knee or hip replacement surgery is that they could improve patient mobility; however, that necessarily does not improve the quality of life or eliminate the need for revision surgery, especially in young patients [186]. Several studies indicate that patient-specific implants could overcome many challenges in traditional hip and knee replacement approaches [187,188,189]. For example, in a 2012 study reported by Murr et al., open-cellular structures of Ti-6Al-4V and Co-29Cr-6Mo implants were printed using the EBM technique yielding a porous, monolithic, and patient-specific product with superior bone-ingrowth capabilities [190]. In a 2016 study by Arabnejad et al., a porous, titanium femoral stem 3D-printed using SLM exhibited reduced stress-shielding, attributed to the optimal relative density distribution resulting in natural bone-like mechanical properties [191]. Surface modifications are usually performed on traditional implants to improve the bioactivity of the bone-metal interphase. AM can incorporate micro resolution surface architectures during printing, which can partially or fully replace an additional surface treatment. Noel and colleagues had compared a Ti-6Al-4V hip stem component with proprietary AM Osteo Anchor architecture against plasma-sprayed Ti coating that is prevalently used. After implanting in animals, it was observed that proprietary AM Osteo Anchor architecture exhibited improved primary fixation and bone-ingrowth. Since hip and knee replacement is one of the widely performed implantations, the single-step, patient-specific, and mechanically superior implant manufacturing offered by AM reduces the need for revision surgery and further improves patient mobility compared to traditional practice.

#### 4.5.5. Sternum Implant

Sternum repair is often performed after open-heart surgery [192]. However, total sternum replacement is an uncommon surgery, and no streamlined protocol exists clinically [193]. One of the earliest reported case studies of a sternum replacement surgery using AM was from a 2013 study by Turna et al. A female patient with metastatic breast carcinoma extended to sternum received a sandblasted SLS printed titanium sternum. In another 2016 study, a similar approach was adopted, where an additively-manufactured half sternum with three adjacent ribs was printed with costo-sternal joints, which mimicked natural movements [194]. In a 2018 publication by Anton et al., a 3D-printed titanium sternum was implanted in a 70-year-old female patient with a sternal tumor. Dislocation or migration is one of the risks associated with a surgery of this degree, and precise 3D printed surgical guides were used in pre-operative planning, which helped the surgeons plan the number and location of fixtures. This study presented a novel multidimensional approach for chest wall reconstruction, which could be advantageous, especially in traumatic repairs [164].

#### 4.5.6. Spinal Implant

Spine implantation is a billion-dollar market with a lot of big players, such as Renishaw and Medtronic, involved. Additive manufacturing is the most preferred choice of manufacturing due to the reduced cost and patient-specificity [195]. Congenital or traumatic medical conditions, such as degenerative disc disease, herniated disc, spondylolisthesis, spinal stenosis, and osteoporosis, could result in spinal implantation to correct the intervertebral height of the patient [196]. Three-dimensional printing can be employed for pre-operative planning, screw guides, osteotomy guides, tumor repair, bio-tissue manufacturing, and education [197]. One of the reported applications of 3D printing in spinal implantation was of a 14-year-old girl with hemangioendothelioma. The patient received a custom-made Ti-6Al-4V reconstruction cage for the T9 vertebral body, which accommodated the kyphoscoliotic nature. The study also reports that the patient had no complications and exhibited bone integration in the 6th-month follow-up [198]. In a 2016 study by Xu et al., a 12-year male patient with C2 Ewing sarcoma received an EBM-printed titanium alloy C2 self-stabilizing artificial vertebral body (SSAVB) with personalized wings. In the 12th follow-up, the patient had improved neurological function with a Japanese Orthopaedic Association (JOA) score of 16/17 alongside improved osteointegration [199]. Another clinical case study of a 3D-printed spinal implant was reported by Mobbs et al. in 2019, where a 34-year-old male patient with spondylosis caused by a congenital anomaly in L5/S1 of anterior lumbar interbody fusion (ALIF) received a DMLS printed Ti-6Al-4V corrective implant with angled holes. The design aspect of this implant favored an easy insertion with press-fit as per the report, and the patient did not suffer from any intra/postoperative complications with reduced radiculopathy pain and early osteointegration [200]. Unlike an over-the-shelf (OTS) implant, the custom printed spine implants were observed to mimic the natural function of the replaced bone, thereby inducing superior osteointegration and reduced inflammatory response [198,201].

#### 4.5.7. Mandibular Implant

Mandibular implants are widely used to reconstruct the jaw due to diseases, such as osteonecrosis, inflammation, or cancer, or an accident. In addition, mandibular augmentation is a burgeoning cosmetic surgery that increases the demand for mandibular implants. The mandible has significant physiological functions, such as craniofacial development, mastication, breathing, and deglutition [202]. In traditional clinical practice, the reconstruction was performed using bone grafts obtained from the tibia, rib, and iliac crest [203]. However, this approach could not yield a perfect aesthetic fit. Three-dimensional metal printing is a perfect solution to construct a load-bearing implant with a good aesthetic fit. In addition, the patient satisfaction post-implantation was found to be significantly higher with 3D printed mandibular implants compared to conventional approaches, such as titanium reconstruction plates and autologous grafting. Furthermore, the facial symmetry can be restored, and AM mandibular implants offer maximum mouth opening and occlusal function compared to conventional approaches [204]. In the study by Yan et al., four patients with ameloblastoma and squamous cell carcinoma were recruited to receive a custom EBM-printed titanium mandibular implant. The test group had a mean operative time of 130 min and an average follow-up of 12 months, with normal maximum mouth opening ranging from 25 to 40 mm. All patients had a satisfactory postoperative dental rehabilitation when considering criteria, such as speech, chewing pain, occlusal force, swallowing, or eating difficulty [204]. In another case study by Park et al. from 2020, a 53-year-old male patient suffering from osteoradionecrosis received an SLM-printed titanium implant with premounted dental fixtures. Here, the titanium mandible was not immediately operated on for dental prosthesis installation due to the risk of infection; however, the installation was performed after proper osseointegration of the mandible [205].

#### 4.5.8. Dental Screws and Prostheses

Dental implantation is comparatively less complicated and the most prevalent implantation surgery with a wide scope for additive manufacturing [206]. The results can be easily analyzed, post-surgical complications can be identified at early stages, and any corrections can be implemented effortlessly compared to other implants. According to a 2019 review, nearly 1322 articles are published based on 3D-printed dental implants, pointing towards the scope of AM in dentistry. Most commonly used are titanium/alloys, nickel alloys, and cobalt-chromium; however, nickel alloys are sidelined in current clinical practice due to allergic reactions it triggers [207]. Milling was one of the conventional techniques used in CoCr dental prosthesis manufacturing; however, metal shrinking is a common shortcoming with this technique, which can be eliminated in 3D printing [208,209]. Several clinical references cite AM in endodontics, prosthodontics, maxillofacial surgery, periodontal surgery, orthodontics, and dental implantation [207]. In a study conducted at McGill University, Montreal, it was observed that SLM-printed removable partial dentures are more favorable than cast metal dentures from patient convenience and satisfaction standpoints. The higher mimicking resolution offered by AM helps in improved comfort, mastication, speech, and oral hygiene [210]. A 2016 study by Samy et al., with 3-year long patient follow-up, elucidates the performance of DMLS-printed Ti-6Al-4V implants. In this study, 86 volunteers received a total of 110 implants with the same number of prosthetic restorations using single crowns, and at the end of the third year, there was a 94.5% implant survival rate and 94.3% crown success rate. The researchers concluded that ‘AM is a successful clinical option for the rehabilitation of single-tooth gaps in both jaws [211].’ Dental prostheses were often reconstructed using a metal-ceramic or all-ceramic composition. However, ceramic is prone to chipping or fracture, which could comprise the prosthesis and, therefore, AM single crowns or fixed-dental prosthesis (FDP) made from Co-Cr or titanium alloys are widely preferred. Hesse et al., in their 2020 review paper, identify that SLS is widely preferred for CoCr printing, while EBM is the preferred method for the construction of titanium dental prosthesis [212]. However, the same review criticizes the lack of standardization and regulation in the AM of dental prostheses, which shall be attributed to the early hiccups in this technology.

#### 4.5.9. Bone Scaffold

A bone scaffold is identified as a three-dimensional matrix made of a biocompatible material that can mimic the working of actual bone, i.e., attachment and proliferation of osteogenic cells [213]. Ghassemi and colleagues, in their 2018 review paper, identified a few criteria for bone scaffolds, of which the mechanical strength, especially during the amelioration phase and architecture mimicking capability, are two important parameters that emphasize the need for a better metal scaffold [213,214]. However, AM had already solved the mimicking of complex bone architecture to an extent with its different lattice structure adaptations and is on the verge of perfecting the natural bone structure [215,216]. One of the early studies of the natural bone mimicking scaffold was reported by Wu et al. in 2008, where hot isostatic pressing was employed to 3D print NiTi and Ti scaffolds with improved super hydrophilicity and hydroxyapatite deposition. One of the recent clinical studies on a live model was reported in 2020 by Crovace et al., in which an EBM-printed Ti-6Al-4V porous scaffold was implanted in a sheep model. The study elucidated the scope of 3D printing in repairing large bone defects. The implant had the optimal load-bearing capability while retaining its functional capabilities [217]. The adaptation of AM metallic bone scaffolds is yet to be clinically translated; however, it shows a promising future in large bone defect repairs.

**Table 4:** Summary of AM implantations, type of processing, the material used, and additional clinical citations.

S.No	Type of Implant	Purpose of Implant	Process	Material	Details of Case Study	Parameters/Specifications	Reference
1.	CRANIOFACIAL	To protect the brain and alleviate psychological affliction caused by the bone defect and restore the patient’s appearance and psychological stability.	EBM	Ti6Al4V ELI	A 38-year-old patient was referred to a craniofacial surgeon with a large cranial defect in the left parieto-temporal area. Cranial reconstruction surgery was performed.	Powder size—50–100 μm, Implant thickness—1.25 mm, Pore size diameter—700 μm Strut size—800 μm	[218]
			DMLS	Ti6Al4V ELI	A 22-year-old male patient had a large post-trauma defect in the right frontal bone. Reconstruction of the cranial defect was required to restore the structural integrity of the skull and the patient’s facial aesthetics.	Cranial replacement area—12.5 × 8.4 cm^2^	[219]
			DMLS	Ti6Al4V ELI	A 28-year-old male patient had a large post-trauma defect in the right frontal bone. Reconstruction of the cranial defect was required to restore the structural integrity of the skull and the patient’s facial aesthetics.	Bone defect area—13.5 × 9.4 cm^2^, Total weight of prosthesis—82 g, Thickness—2–3 mm	[220]
			EBM	Ti6Al4V ELI	A 7-year-old girl had a huge frontonasal bone defect with consequent hypertelorism. Reconstruction of the cranial defect was required to restore the structural integrity of the skull and the patient’s facial aesthetics.	Powder size—45–100 μm, Implant weight—12.20 g, Implant thickness—7 mm, Pore size diameter—2mm	[221]
			EBM	Ti6Al4V	Three female patients (tumor—one patient, trauma—two patients) were chosen for the study. Reconstruction of the cranial defect was required to restore the structural integrity of the skull and the patient’s facial aesthetics.	Patient 1 defect size—12 × 14 cm^2^, Patient 2 defect size—14 × 11 cm^2^, Patient 3 defect size—15 × 15 cm^2^	[222]
			EBM	Ti6Al4V	A 27-year-old woman with a wide cranial vault lacuna in the upper part of the skull and slightly crossing the sagittal plane underwent reconstruction surgery to restore the shape and function of the cranium.	-	[173]
2.	MAXILLOFACIAL	To achieve correct shape of orbital wall or jaw and reconstruction followed by resection of the tumor region.	DMLS	Ti6Al4V ELI	The patient was a 67-year-old male who had been in a severe accident. Reconstructive treatment was performed to achieve anatomically correct shape of the orbital wall and appearance of the eye symmetry.	Thickness—0.4 mm, Hole size—3 mm, Hole size (screw)—2 mm	[223]
			EBM	Ti6Al4V ELI	Tumor treatment—The mandible section with the tumor on the patient’s left side was removed and replaced by mirroring the healthy right mandible.	Powder size—50–100 μm, Offset thickness—2 mm, Mesh size—0.4 mm,	[224]
			EBM	Ti6Al4V ELI	A 40-year-old patient underwent a multilocular radiolucent lesion on the right posterior mandible. Reconstruction of the discontinuous mandible defect was performed.	-	[225]
			SLM	Ti6Al4V-Grade 2	The 50-year-old patient presented maxillary epidermoid carcinoma history with nasal affection addressed two years ago by a total maxillectomy and total nasal amputation. Nasal reconstruction was performed.	Thickness—0.4–0.7 mm, Pore dimension—860–1500 μm	[226]
			SLM	cT4N1M0	A 53-year-old male suffered osteoradionecrosis due to the radiation therapy after squamous cell carcinoma resection of attached gingiva in the left mandible. The reconstruction plate was fixed.	-	[205]
3.	DENTAL IMPLANT	To restore the function of the tooth or jaw affected due to tumors or accidents.	SLS	Ti6Al4V	16 patients with possible dental repair were voluntarily recruited for the clinical study.	Powder size—25–45 μm, Diameter—2.7 mm, Length—10 mm	[227]
			DLMS	Ti6Al4V	15 patients, 8 males and 7 females (age 39–55), were selected for the study based on the possibility of a dental repair.	Powder size—25–45 μm, Cylindrical implant: alveolar apex—3–5 mm	[228]
			DMLS	Ti6Al4V	A 17-year-old male patient who sustained an injury to the anterior maxillary region leading to loss of upper front teeth along with bone was presented in this case study.	-	[229]
			DMLS	Ti6Al4V	44 males, 38 females, age range 26–67 years were voluntarily recruited for the study.	Laser wavelength—1054 nm, Laser power—200 W, Scanning rate—7 m/s, Laser spot size—0.1 mm, Powder size—25–45 μm	[211]
			DMLS	Ti6Al4V	39 males and 31 females, aged 62–79 years with dental repair were voluntarily enrolled for the study.	Laser wavelength—1070 nm, Laser power—50/W	[230]
4.	SPINAL IMPLANT	Degenerative diseases, fractures, and other disorders can lead to the functional loss of the spine. Spinal fixation or spinal reconstruction can retain the function of the spine after the resection of the affected area	DMLS	Ti6Al4V Grade 5	A 45-year-old man presented with neck and left arm pain combined with shoulder weakness. Imaging revealed significant destruction of the C3-C5 vertebrae, and chordoma diagnosis was confirmed by biopsy.	Laser power—200 W, Laser spot diameter—55 μm, Layer thickness—20–40 μm	[231]
			DMLS	Ti6Al4V ELI	A 16-year-old boy had a severe kyphotic deformity of the thoracic spine resulting from neurofibromatosis type I.	Pore size—500–600 μm	[232]
					A 63-year-old woman with progressive paralysis due to a severe cervicothoracic dissociation.	Implant width—10 mm, Depth—5 mm, Height—8 mm	
			EBM	Ti6Al4V	9 patients (2 males and 7 females) were included in the study with a mean age of 31.4 years (12 to 59 years) for reconstruction following resection of the primary tumors of the upper cervical spine.	Powder size—45–100 μm	[233]
			FDM	Ti6Al4V	A 12-year-old patient suffering from congenital scoliosis due to an L1 hemivertebra underwent a corpectomy and stabilization surgery from Th9 to L4.	-	[234]
5.	FOOT/HAND IMPLANT	Foot or hand implants are used for reconstructing the defective/fracture/tumor-affected bone.	EBM	Ti6Al4V	A 40-year-old man presented with two-week-long paresthesia in his right hand and limited forearm rotation due to dislocation of the radial head attributed to a traumatic injury during childhood.	Length of the implant—15 cm, Weight—67 g, Pore size—700 and 1500 µm	[235]
			EBM	Ti6Al4V ELI	A 23-year-old soldier was diagnosed with a calcaneal desmoplastic fibroma. Reconstruction surgery was performed for the bone tumor calcaneus.	Length of the implant—63.5 mm, Height—43.2 mm, Weight—104 g	[236]
			EBM	Ti6Al4V	A 71-year-old man presented with a destructive and highly metabolic lesion in the right calcaneus. A total calcanectomy was performed, and the defect was reconstructed with 3D printed titanium calcaneal prosthesis.	Implant weight—280 g	[159]
			EBM	Ti6Al4V ELI	3 patients (one male and two females) had undergone surgery for oncological diagnosis, and reconstruction surgery was performed.	-	[237]
6.	PELVIC IMPLANT	Pelvic implants provide support or replace the weaker bones due to arthritis, tumor, or fracture.	SLM	Ti6Al4V	A 65-year-old man presented with expansile osteolytic destruction at the anterior column of the left acetabulum. Pelvic tumor resection and prosthetic reconstruction of the bone defect were planned in the study.	-	[166]
			EBM	Ti6Al4V ELI	7 patients (3 males and 4 females) were chosen for the study based on existing pelvic/hip morbidity. Pelvic reconstruction was performed.	-	[237]
			EBM	Ti6Al4V	13 patients were chosen for the study, of which 3 patients had total hip replacement surgery, and 4 patients had pelvic resection surgery.	-	[238]
			EBM	Ti6Al4V	A total of 35 patients (20 males and 15 females) underwent resection of pelvic tumor and reconstruction using 3D printed endoprostheses.	-	[239]
			EBM	Ti6Al4V	30 patients were involved in the study for trabecular bone reconstruction for early osteonecrosis of the femoral head.	Power of E-beam—3000 W, Diameter of electron beam—180 µm, Melting speed—55 to 80 cm^3^/h, Degree of vacuum work area <1 × 10^−4^ mbar	[185]
7.	STERNUM IMPLANT	Sternum implants protect the heart, lungs, and chest blood vessels in people with a compromised sternum. The tumor-affected sternum can also be reconstructed using sternum implants.	EBM	Ti6Al4V	A 57-year-old man suffered from minor thoracic trauma because of prolonged chest pain and chest wall tumor in the chondrocostal junction. A segment of the sternum was replaced to restore the function.	Implant size—147.36 × 180.14 × 128.30 mm^3^	[194]
			DMLS	Ti6Al4V	A 70-year-old woman was affected by the sternal tumor and subtotal sternotomy. Resection of the sternal body with the adjacent sternocostal cartilage was performed.	Weight of the implant—53.5 g, Size of the implant—170 × 60 × 105 mm	[164]
			EBM	Ti6Al4V	A 19-year-old woman presented with anterior chest wall instability and paradoxical movement with respiration. Reconstruction after anterior chest wall resection was performed.	-	[240]
			SLM	Ti MG 1	A 70-year-old male, with a right anterior pectoral mass approximately 10 by 9 cm was presented in the study for chest wall resection following wide local excision for bone tumor.	-	[241]
			SLM	Ti6Al4V ELI	A 62-year-old female was presented with a mass located on the chest wall associated with foul smelly drainage. Reconstruction after chest wall resection was performed.	The thickness of the implant—2–3 mm, Weight—160 g	[242]

## 5. Future Scope and Challenges

Personalized medicine is expected to be the next big revolution in the medical industry, and additive manufacturing has the best potential to keep up with the expectation of personalized implantation. The degree of freedom, precision in designing, and smart material integration are some of the salient features of AM. The versatility of materials employed for 3D printing makes it one of the major biomaterial or implant manufacturing processes. The advancement in computation and electronics enabled the democratization of 3D bioprinters, allowing more people to get hold of 3D printing equipment, which would considerably reduce the cost of AM of implants and crank up the technological advancements [243]. In the current generation of 3D printing, only bioinert or bioactive implants have been experimented with clinically; however, the next-generation AM focuses on bio 3D printing tissues and organs, which could be a game-changer in the medical industry.

Even though the future of AM looks promising, a few challenges need to be addressed before fully relying on AM. Certainly, the biomimetics of implants are improved compared to the conventional approach; however, this technology is still in its incubation phase. A few technological challenges include microstructural anisotropy, inferior mechanical properties due to void formation, stair-stepping affecting the external surface features, and limitation in scaling up at an industrial scale. Even the Food and Drug Administration (FDA) is still working on norms and regulations for making AM a safe clinical practice [244].

## Figures and Tables

**Table 2:** Tensile strength and elastic modulus of human bone, and common biomaterials (conventional vs. AM) [17,19,20,27,29,32,33,49,132,139,140].

Materials	Tensile Strength (MPa)	Elastic Modulus (GPa)
Natural Bone		
**a.** **Tibia**	140	18.1
**b.** **Femur**	121	17.2
**c.** **Radius**	149	18.6
**d.** **Humerus**	130	17.2
**e.** **Cervical**	3.1	0.23
**f.** **Lumbar**	3.7	0.16
Conventional Metals/Alloys		
**a.** **CP Ti**	785	105
**b.** **Ti-6Al-4V**	970	110
**c.** **Ti-6Al-7Nb**	1024	105
**d.** **Stainless steel 316L**	460–950	200
**e.** **Co-Cr alloys**	655–1896	210–250
AM Porous Metals/Alloys		
**a.** **CP Ti**	78–245.5	5.5–8.5
**b.** **Ti-6Al-4V**	64–409	3.8–7.8
**c.** **Ti-6Al-7Nb**	105	1.2–4-5
**d.** **Stainless steel 316L**	300	0.15–0.12
**e.** **Co-Cr alloys**	60–150	20–25

## Data Availability

Not applicable.
